# Editorial: Early identification and prevention of suicidal crisis in children and young people

**DOI:** 10.3389/frcha.2024.1507862

**Published:** 2024-12-11

**Authors:** Emma Ashworth, Saskia Mérelle, Pooja Saini

**Affiliations:** ^1^ School of Psychology, Faculty of Health, Liverpool John Moores University, Liverpool, United Kingdom; ^2^ Research Department, 113 Suicide Prevention, Amsterdam, Netherlands

**Keywords:** suicidal crisis, children and young people, early intervention, suicide prevention, clinical intervention, public health

**Editorial on the Research Topic**
Early identification and prevention of suicidal crisis in children and young people

## Introduction

Suicide is a major public health issue globally, ranking as the third leading cause of death among children and young people (CYP) worldwide (1). Concerningly, suicide rates among CYP have been increasing in recent years, especially in Western countries (2).

Suicidal crisis, defined as a spectrum ranging from passive thoughts about death to specific suicidal ideation with a clear intent or plan, has been identified as a key risk factor for future suicide attempts (3). Suicidal ideation often precedes a suicide attempt, with over one-third of adolescents in suicidal crisis going on to attempt suicide (4). Additionally, the more pervasive the suicidal crisis, the more likely the individual is to attempt suicide (5), with 80% of people who die by suicide seeking help for suicidal crisis at least once in the year before their death (6).

Thus, early identification of CYP experiencing suicidal crisis and the implementation of effective prevention strategies are vital in preventing future distress and later suicide attempts. However, while numerous studies have been conducted into prediction and prevention of suicidal thoughts and self-harm, relatively little is known about suicidal crisis as a discrete stage, particularly in CYP. Indeed, there is much work to be done to identify:

1. Risk and protective factors for suicidal crisis in CYP
2. Novel approaches to reducing suicidal crisis, including universal and targeted programmes
3. Effective early intervention through prevention
4. How to predict, prevent, and support suicidal crisis among vulnerable populations

This Research Topic aims to bring together a collection of works contributing to the evidence base in these four domains. This introductory Editorial outlines their contributions and the ways in which they advance the field. The six original articles focus on three key themes, discussed in turn below.

## Predictors and mediators of suicidal crisis in CYP

Risk factors for suicidal crisis are given substantially less attention in the evidence base than factors associated with suicide attempts or completed suicides. Two contributions to this Special Issue focused on examining the interactions and interrelationships between risk factors, as well as the roles of mediators, both in Chinese adolescent populations.

Zhong et al. found both poor sleep quality and high impulsivity directly linked with suicide ideation in school students, with impulsivity also acting as mediator between sleep quality and suicide ideation. While research into the relationship between sleep and suicide ideation has previously been conducted, findings have been mixed; however, the mediating role of impulsivity may help to explain incongruent findings. Zhong et al. conclude that impulsivity is a critical factor in the relationship between sleep and suicidal ideation, highlighting new avenues for early intervention.

Wang et al. explored risk factors in the family domain, finding that adolescents with strong perceptions of conflict with parents and low parent-child attachment were at high risk of suicide ideation. Furthermore, parent-child attachment played a mediating role between parental conflict and suicide ideation, emphasising the key protective role of parental relationships.

## Public health interventions

Recently, there has been a shift in how suicide prevention is viewed and addressed, with a new Lancet Series calling for a public health approach that uses universal interventions to address the social determinants of suicide at a population level (7). It is argued the greatest reductions in suicide are most likely to be achieved through upstream public health measures aimed at the whole population, rather than targeting individuals at heightened risk (8).

Aoun et al.’s scoping review examined the effectiveness of both universal and selective interventions in reducing suicidal crisis among adolescents, finding some promising interventions that incorporated technology. Indeed, mobile apps provide increased accessibility to therapeutic interventions precisely when needed, while social media can help improve help-seeking behaviour, with adolescents using these platforms to confide in their friends, seek support, and share experiences.

However, social media can be a double-edged sword, with potential benefits being overshadowed by unregulated platforms sharing content that may cause distress or lead to imitative behaviour. Robinson et al. used a Delphi approach to identify steps the social media industry and policymakers could take to improve online safety. Study findings were mixed, reflecting the complexity associated with trying to minimise the risks whilst capitalising on the benefits. Participants agreed that suicide-related content should be restricted, companies should have clear policies covering content promoting self-harm or suicide, and governments should require schools to educate students on safe online communication.

## Clinical interventions

While the utility of a public health approach is evident, there is still a clear need for effective clinical interventions for CYP already experiencing suicidal crisis. Looijmans et al. conducted a qualitative study with young adults with experience of suicide ideation in the Netherlands, exploring their perspectives regarding CYP suicide prevention. Important factors in clinical services included the therapeutic alliance, openly and meaningfully discussing suicidal thoughts with healthcare professionals, reduced waiting lists, transitional care between healthcare facilities, and the introduction of more peer specialists.

Within the Dutch clinical field of youth mental health care, a subgroup with severe and chronic suicidal behaviour is being recognised. Van de Koppel et al. provided a case description of an 18-year-old female who died by suicide, as part of a psychological autopsy study. Findings showed repeated admissions to the crisis unit following suicide attempts, but only temporary reductions in suicidal behaviour with each admission. The care provided became increasingly characterised by safety management, leaving the treatment of underlying problems unaddressed. The authors argued for the need to treat suicidality as a transdiagnostic phenomenon, implementing treatment plans that promote recovery and growth rather than focusing solely on preventing fatal outcomes.

## Moving forward

The articles in this Research Topic make important contributions to our understanding of suicidal crisis in CYP. However, further work is still required to better understand suicidal crisis as a discrete stage in the suicidal process. Greater insights would allow for identification of effective ways of intervening early, in line with an upstream, public health approach, as well as more effective clinical care for individuals already experiencing suicidal crisis.
